# Henipavirus in *Pteropus vampyrus* Bats, Indonesia

**DOI:** 10.3201/eid1204.051181

**Published:** 2006-04

**Authors:** Indrawati Sendow, Hume Ernest Field, John Curran, Darminto, Chris Morrissy, Greer Meehan, Tim Buick, Peter Daniels

**Affiliations:** * Research Institute for Veterinary Science, Bogor, Indonesia;; † Department of Primary Industries and Fisheries, Brisbane, Queensland, Australia;; ‡ Australian Quarantine and Inspection Service, Broome, Western Australia, Australia;; § Assessment Institute for Agricultural Technology, Malang, Indonesia;; ¶ Commonwealth Scientific and Research Organization Australian Animal Health Laboratory, Geelong, Victoria, Australia;; # Biosecurity Australia, Canberra, Australian Capital Territory, Australia

**Keywords:** Hendra, Nipah, Henipavirus, Emerging, Bats, Pteropus, Flying foxes, Reservoir, Indonesia

**To the Editor:** The emergence of Nipah virus (NiV) in Malaysia in 1999 resulted in 265 known human infections (105 fatal), widespread infection in pigs (with >1 million culled to control the outbreak), and the collapse of the Malaysian pig export market (*1*). As with the closely related Hendra virus (HeV) that emerged in Australia in 1994 and caused fatal disease in horses and humans (*2*), bats of the genus *Pteropus* (commonly known as flying foxes) were identified as the major reservoir of Nipah virus in Malaysia (*3**,**4*). This report describes a serologic survey of *Pteropus vampyrus* in neighboring Indonesia.

We nonrandomly sampled 106 *P. vampyrus* bats from market sellers on the Indonesian islands of Java and Sumatra during a 12-day period from July 23 to August 3, 2002 (Figure). Bats were typically caught locally by sellers. Screening by indirect enzyme-linked immunosorbent assay with inactivated NiV antigen was done at the Research Institute for Veterinary Science in Bogor, Indonesia. Virus neutralization tests (VNT) with NiV and HeV were performed under biosafety level 4 conditions at the Commonwealth Scientific and Research Organization (CSIRO) Australian Animal Health Laboratory in Geelong, Australia. The gold-standard (*6*) VNT results are presented here; a neutralizing titer >5 was considered positive.

**Figure:**
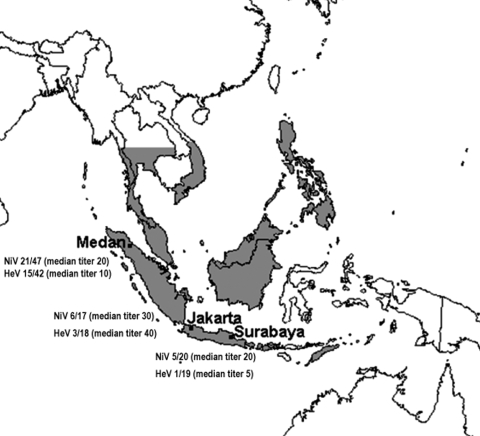
Geographic range of *Pteropus vampyrus* (*5*) and proportion of bats whose sera neutralized Nipah virus (NiV) and Hendra virus (HeV) at each location. Numbers are given as the ratio of the number of positive samples to the total number of positive and negative samples (excluding bats in which a toxic reaction precluded a definitive test outcome and bats that had inadequate samples for neutralization testing).

Serum samples from 32 bats neutralized NiV (median titer 20, range 5–160), samples from 52 bats did not, and samples from 20 bats caused toxic reactions in the cell sheet at dilutions <10 (n = 7), <20 (n = 9), or <40 (n = 4), precluding a definitive test outcome. Two bats had inadequate samples for NiV VNT. Samples from 19 bats neutralized HeV (median titer 10, range 5–80), samples from 60 bats did not, and samples from 27 bats caused toxic reactions at dilutions <10 (n = 18), <20 (n = 7), or <40 (n = 2), precluding a definitive test outcome. Of the 70 bats whose samples had a definitive outcome in both tests, 11 neutralized NiV only, 1 neutralized HeV only, and 17 neutralized both viruses. Of these 17 bats, 14 samples had a higher titer to NiV than to HeV, 2 had identical titers to each virus (5 and 10), and 1 had a higher titer to HeV (40) than to NiV (20). Infection was attributed to NiV in 25 bats (11 whose samples neutralized only NiV and 14 whose sera neutralized both viruses but had a higher titer to NiV), a prevalence of 35.7% (95% confidence interval [CI] 24.6%–48.1%). Infection was attributed to HeV in 2 bats (1 had a HeV titer of 5 and no NiV titer, and the second had a HeV titer of 40 and a NiV titer of 20), a prevalence of 2.9% (95% CI 0.3%–9.9%).

The detection of antibodies that neutralized NiV at all 3 sampling locations indicates that infection with NiV (or a cross-neutralizing virus other than HeV) is widespread in *P. vampyrus* in Sumatra and Java. These findings, in conjunction with earlier findings in peninsular Malaysia, suggest that NiV infection is likely to be found in *P. vampyrus* across its entire range (Figure). Recent satellite telemetry studies showing regular *P. vampyrus* movements from Malaysia to Sumatra and Thailand also support this contention (*7*). Additionally, experience with HeV in Australian flying fox populations suggests that where susceptible flying fox species share communal roosts, evidence of infection is seen in in-contact species (*8*). Therefore, NiV (or a Nipah-like virus) infection probably occurs in other *Pteropus* species whose geographic distributions overlap or abut that of *P. vampyrus*. This contention is supported by the positive NiV serologic findings in *P. lylei* in Cambodia in 2002 (*9*) and *P. giganteus* in India (J. Epstein et al., unpub. data) and Bangladesh (*10*).

Infection was attributed to HeV in only 2 bats. The finding of 2 true HeV-positive bats in Medan and Jakarta would require sporadic HeV infection in a population in which NiV infection predominates or, alternatively, nomadic movement of animals from a population in which HeV circulates. Given the equivocal HeV titers in the 2 bats, these results are likely false positives.

The findings indicate that NiV or an unidentified Nipah-like virus is endemic in *P. vampyrus* in Indonesia. Further interpretation is limited by the nonrandom sample, the <100% specificity of the VNT, and the inability to corroborate serologic results by virus isolation or polymerase chain reaction (tissue collection was not permitted by Indonesian wildlife authorities).

Similar serologic findings are likely in overlapping *P. vampyrus* populations and possible in overlapping populations of other *Pteropus* species. Further research is needed to explain the geographic extent of NiV infection in flying foxes and the nature and stability of the interface between HeV and NiV, and to investigate the possible presence of other cross-neutralizing henipaviruses.
